# A Cross-Regional Scheduling Strategy of Waste Collection and Transportation Based on an Improved Hierarchical Agglomerative Clustering Algorithm

**DOI:** 10.1155/2022/7412611

**Published:** 2022-03-30

**Authors:** Zeming Wei, Chufeng Liang, Hua Tang

**Affiliations:** School of Computer Science, South China Normal University, Guangzhou 510632, China

## Abstract

The disposal of municipal solid waste (MSW) is based on the divide-regional operation model at present, which brings management convenience. However, due to the constraints of the division between the operating regions, there are problems such as inflexible vehicle scheduling and low efficiency. Changing this mode will put pressure on management. It is necessary to break the constraint of the region and form a fully automatic operating scheme without too much manual management. First, the initial clusters are formed by considering the distance in the initial allocation module. Secondly, through the type labeling and reallocation module, the single big data set is transformed into multiple small data sets by considering the allocated amount of garbage and the carrying capacity of garbage vehicles. Then, this work proposes the improved hierarchical agglomerative clustering (IHAC) algorithm and the garbage collecting path planning (GCPP) algorithm to realize the intelligent allocation of waste and scheduling route planning of garbage vehicles. Finally, through simulation by real example and comparative analysis, the advantages of the proposed scheme are discussed. The results show that the proposed scheme is more effective than the original scheme and other advanced methods, which can provide decision support for the scientific and intelligent collection and transportation of MSW.

## 1. Introduction

**Figure 1:**
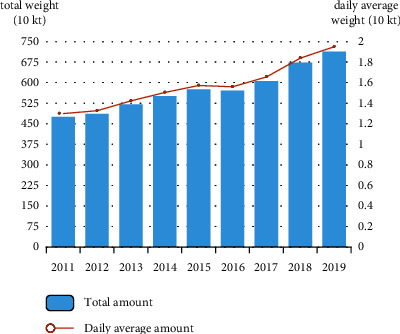
The total amount of MSW disposal in X city every year.

With the improvement of people's living standards and the acceleration of urbanization, the amount of municipal solid waste (MSW) is increasing year by year. As shown in Figure 1, the MSW disposal amount of a certain city in China (for the sake of confidentiality, we define the name of the city as X) reached 7.124 million tons in 2019, an increase of 6.05% over the previous year. According to the prediction of “Analysis Report on Domestic Waste Industry in China in 2016,” the production of MSW will reach 223 million tons in 2021. There is an urgent need for scientific and reasonable disposal of MSW. Waste treatment has become an urgent issue in the comprehensive improvement of the urban environment. How to properly dispose of domestic waste has become a problem faced by all countries in the world.

The disposal of MSW must consider the cost of the operation. Collection, transportation, and storage of MSW account for 60%–80% of the total cost of MSW treatment [1]. Therefore, the various processes of garbage collection and transportation are the key to controlling costs. However, these processes are mainly based on the divide-regional model at present. The division of the administrative districts of city X is used to delineate the various environmental sanitation operation regions. Each region is independent. There are the following problems in this divide-regional operation model:

- High operating cost: the divide-regional operation model restricts the various processes of collection and transportation of MSW. The garbage collected from a particular street in a region can only be compressed at a garbage transfer station on the street in that region. This scheme also specifies which terminal treatment plant will handle the garbage from each region. These restrictions result in high costs.
- Unreasonable garbage allocation: since each region specifies which terminal disposal plant will handle its garbage, each terminal disposal plant forms a quota limit on the amount of garbage entering that plant for each region. In the current environmental sanitation operations in city X, there are frequent incidents of unreasonable garbage allocation and excess garbage amount, which will further threaten the disposing capacity of the garbage treatment plant, and these incidents are also caused by the constraints of this model.
- Inflexible garbage vehicle scheduling: the divide-regional operation model restricts the collection and transportation of garbage vehicles to a fixed region and lacks flexibility in scheduling. Additionally, the scheduling of garbage vehicles is mainly manual, resulting in huge human resources and vehicle operation consumption.

By analyzing the above problems in the divide-regional operation model, we can find that the constraints of this model cause these problems, and it is necessary to break the constraints of this model. The ideal approach would be to no longer set constraints on the region, which would greatly reduce the operating costs and allow for flexible vehicle scheduling. All processes of environmental sanitation operations would not be restricted by regions. However, this would also bring management difficulties [2]. Therefore, it is necessary to plan the operation process and vehicle scheduling to allocate garbage according to the amount accurately without setting region restrictions.

The route planning of vehicle is a vehicle routing problem (VRP) [3]. In environmental sanitation operations, the vehicle is the garbage vehicle. Therefore, not only is the road distance factor considered, but also the garbage amount factors should be taken into consideration, such as the maximum carrying capacity of the vehicle and the daily waste disposal amount of the terminal disposal plant. Therefore, this problem is converted into a capacitated vehicle routing problem (CVRP). In actual operations, the collection and transportation of garbage must also consider the geographic location of the relevant sanitation components. Since the early sanitation components have been built, their addresses may be unreasonable, and replanning their reasonable addresses requires many human resources, material resources, and time. Therefore, it is necessary to conduct an in-depth study on the operation path planning and modeling of garbage vehicles. Capacitated vehicle routing problem is classified as an NP-hard problem, and normal optimization algorithms cannot solve it. This work discusses a new approach to solve this problem: using the proposed improved hierarchical agglomeration clustering algorithm and greedy search algorithm to solve the global optimal route solution.

In addition, with the development of smart cities and the technologies in environmental sanitation management and control, some urban environmental sanitation command centers have been able to obtain the working status of various sanitation components in real time, such as the information on the amount of garbage currently owned by each garbage collection point and terminal disposal plant, as well as the current garbage carrying amount of each sanitation vehicle. These technologies and application systems provide strong support for the optimization and improvement of environmental sanitation operations.

Under the background, this paper proposes a new cross-regional operation scheme based on clustering, which studies the planning of garbage vehicles driving paths and the allocation of waste according to the amount. The combination of operating regions eliminates the regional boundaries that bring many constraints, allowing for flexible waste distribution, effectively reducing the high cost of waste collection and transportation, and avoiding incidents of excessive regional quotas. The data to be clustered are converted from a single large dataset to multiple small datasets through the initial allocation, type labeling, and reallocation modules. Next, this work improves the hierarchical agglomerative clustering [4] and proposes an improved hierarchical agglomerative clustering (IHAC) algorithm to cluster the garbage collection points so that the garbage collection points in each cluster are relatively clustered geographically. The garbage amount of each cluster is close to the nuclear load of the garbage vehicle. The proposed garbage collection path planning (GCPP) algorithm obtains the shortest driving path for garbage collection and transportation of vehicles and realizes the intelligent planning of vehicle garbage collection routes. Finally, according to the actual environmental sanitation situation of city X, this paper makes an empirical analysis of the proposed cross-regional operation model. The results show that the proposed scheme can better solve the defects of the existing operation scheme. Compared with the current operation mode, that is, the divide-regional operation model, garbage vehicles' driving distance and the number of vehicles used are greatly reduced. Simultaneously, this scheme realizes the intelligent scheduling of garbage vehicles, zero consumption of human resources, and the accurate distribution of garbage, which has good feasibility and effectiveness, especially of great significance to the development of the carbon neutralization trend. The contributions of this study mainly involve the following aspects:

- This paper proposes a new model of garbage collection and transportation in environmental sanitation work: cross-regional operation model, which breaks the constraints of the traditional operation model, greatly reduces the operating costs, and realizes the accurate allocation of garbage, as well as solving the problem of the excessive amount of garbage entering some terminal disposal plants.
- CVRP is an NP-hard problem, and the traditional optimization algorithm cannot solve it well. This paper provides a new solution using clustering and the idea of greedy search. The IHAC algorithm comprehensively considers the factors of garbage amount and distance in the various garbage collection and transportation processes. The collection points in each cluster are clustered geographically, and the total amount of garbage in the cluster is close to the maximum carrying capacity of the garbage vehicle, thus maximizing the utilization of garbage vehicle carrying capacity. The IHAC algorithm lays the foundation for planning the optimal driving path of the vehicle.
- The garbage collecting path planning algorithm is proposed. For the clusters formed by the IHAC algorithm, the GCPP algorithm can plan the optimal driving path of garbage vehicles in garbage collection and transportation, which realizes the automatic planning of garbage vehicle operating path.
- This paper establishes the evaluation index system of garbage collection and transportation, and the proposed schemes are compared and empirically analyzed. The real data from the various processes of garbage collection and transportation is used to compare and analyze several schemes, and the effectiveness and feasibility of our method are proved.

The structure of this paper is as follows. In the second section, the current research status of CRVP and vehicle path planning in garbage collection and transportation is introduced.  Section 3 mainly introduces the garbage collection and transportation model and the corresponding mathematical model.  Section 4 mainly illustrates the overall design of the cross-regional operation scheme.  Section 5 focuses on the related algorithm design.  Section 6 mainly analyzes the experimental results. And this section sets up discussions and draws some conclusions.

## 2. Literature Review

Considering the available studies, most domestic and foreign studies on the optimization of garbage collection and transportation in sanitation operations are based on the original model (i.e., the divide-regional operation model), rather than focusing on breaking the regional restrictions in the old model. For one thing, this is because the government's planning policies in this field are for the convenience of management, so few studies will try to do this. For another, it is mainly because the sanitation infrastructure of the city cannot meet the requirements of advanced technological solutions [5]. There is a lack of practical feasibility to break out of the constraints of the old model and to use these cutting-edge technology schemes for optimization. With the development of smart sanitation, this trend is being changed. Some cities have already made plans to break the old framework. Therefore, the research in this paper is at the forefront and is in line with future development.

Most studies are based on the original model to make improvements, mainly including optimizing models and algorithms and combining emerging technologies. The capacitated vehicle routing problem (CVRP) [6] is one of the fundamental problems in combinatorial optimization and has many practical applications in transportation, distribution, and logistics [7]. Pace [8] modeled the transportation problem as a capacitated vehicle routing problem (CVRP) that belongs to the NP-hard problem and used the simulating annealing (SA) heuristic algorithm to solve the model. However, they cannot always identify each address when obtaining the coding value of the geographical location. There is the possibility of deviation to a certain extent to identify each address by using the nearby identifiable addresses. Wang and Mu [9] added the queuing time to the scheduling model of vehicles for optimization and used the particle swarm optimization (PSO) algorithm to solve the model. The PSO algorithm has a fast convergence rate, but the results are random. Ilhan [10] studied CVRP and proposed a new population-based simulated annealing algorithm, which used three different route development operators, namely, exchange, inserting, and reversion regression operators. Most studies are based on the fixed amount of garbage. Li et al. [11] quantified dynamic garbage accumulation by the garbage element and used the Dijkstra algorithm for vehicle route planning. However, they cannot cope with abnormal situations such as the rapid increase of garbage on a certain day. Zhang et al. [12] considered the uncertainty of garbage quantity. They built an optimization model for the route of door-to-door collection and transportation of MSW based on resident time satisfaction. They designed an artificial fish-swarm algorithm (AFSA) to solve the model. Resident time satisfaction should be comprehensively considered from many aspects, whether related to other factors, such as the influence of work service attitude on door-to-door garbage collection. Memon et al. [13] considered the multiple mix zones method to replace parking locations and traffic light/traffic jam places by decorrelation mix zone region. And they presented an improved privacy traffic monitoring system for road network applications via a proposed security scheme, which involved the privacy problem in the allocation of vehicles. Arain et al. [14] introduced multiple mix zones, a new LPPM for map services on smartphones, which did not need to disclose sensitive location information while realizing path planning. Memon et al. [15] presented an efficient pseudonym change strategy with multiple mix zones scheme to provide trajectory privacy protection while implementing road planning and vehicle scheduling network. Some studies focus on introducing emerging technologies, such as the Internet of Things (IoT), deep learning, and GIS technology. Mahmood and Zubairi [16] integrated the scheduling problem into the infrastructure of smart cities to guide the transportation route of garbage vehicles. Using the IoT technology to achieve the scheduling of garbage vehicles requires high coverage of urban infrastructure, which largely tests the level of urban infrastructure. Alqahtani et al. [17] proposed a cuckoo search optimized long short-term recurrent neural network (CLSTRNN), which supports the decision-making of vehicle transportation scheduling. Using this model to analyze data to provide decision-making faces the problems of reliable data sources and data acquisition to some extent. Naveen et al. [18] proposed a cloud-based transportation optimization strategy to achieve optimal load allocation-economic scheduling. Lella et al. [19] used GIS technology to optimize garbage collection and transportation by dividing road networks, and they established feature classes for each region through network analysis. Sharma et al. [20] proposed a novel intelligent fire prediction system based on the Internet of Things. The system improves the machine learning model and combines the Internet of Things technology to predict fire, successfully applying emerging technologies to the prediction model. The use of emerging technologies requires the city's environmental sanitation component configuration to keep up with the application requirements of emerging technologies, which has higher requirements for urbanization. In existing studies, various models, optimization algorithms [21, 22], and emerging technologies are applied to the disposal of MSW. From the perspectives of prediction [23], optimization [24], and decision [25], the problems of waste weight prediction, location and scale of treatment facilities, and vehicle driving route optimization are solved. These optimizations are based on the original operation model, and there are restrictions on the operation region, resulting in very limited optimization space. At present, in environmental sanitation operations, no research has focused on breaking the constraints of the original operation model, which is mainly due to the reasons stated at the beginning of this section. However, with the development of advanced technology, an increasing number of cities have entered the level of intelligence, forming “smart cities” [26]. Cutting-edge technology can help the government departments fully control the environmental sanitation of the city. Therefore, we should pay more attention to breaking the constraints of the old operation mode and use cutting-edge technology to solve the management difficulties brought about, and it will bring a huge optimization space at the same time, including human resources, material resources, and time costs. Therefore, this paper optimizes the waste collection and transportation operation mode in the urban environmental sanitation operation, which is also one of the research goals of this paper.

In our study, the proposed new scheme requires a clustering algorithm. There are six common clustering algorithms at present on the research of clustering algorithm, respectively, *k*-means clustering algorithm [27], which requires preset clustering number, and the results of the clustering are random. Among various optimization algorithms of the *k*-means clustering algorithm, including initial optimization *k*-means++ [28], distance calculation optimization Elkan *k*-means algorithm [29], and optimization Mini Batch *k*-means algorithm under the condition of big data [30], they do not solve the above problems of traditional *k*-means clustering algorithm. Mean-shift clustering algorithm [31] is based on sliding windows, and it does not need to preset the number of clusters. However, it is sensitive to the window size (radius *r*) on the clustering effect. Density-Based Spatial Clustering of Applications with Noise (DBSCAN) [32] also does not need to preset the number of clusters, but its performance is not good when the density of clusters is different. It needs to set the distance threshold and the number threshold, which are difficult to obtain the appropriate value; Gaussian Mixed Model's (GMM) Expectation-Maximization (EX) clustering algorithm is used to adapt to different shapes of data sets and requires a certain understanding of the clustering situation of data sets in advance. The result is not unique; the hierarchical agglomerative clustering (HAC) [33] algorithm does not need to preset the number of clusters. This algorithm is not sensitive to the selection of distance measurement standards. However, these advantages are at the cost of low efficiency and its time complexity is O (n^3^). Graph Community Detection (GCD) [34] algorithm is usually applied to the data with correlation, and the typical application is the social network. In the research scenario of this paper, since the number of clusters is not known in advance, the clustering algorithm used should not need to preset the number of clusters and sensitive parameters in the clustering process. Since the research problem in this paper involves the scheduling of specific routes in the daily work of garbage vehicles, the clustering results should meet the uniqueness. Moreover, the initial data involves the environmental sanitation facilities of the whole city, and the amount of data is large, which has higher requirements for the algorithm's efficiency. Therefore, based on the above clustering algorithms and the requirements of the research scenario in this paper, only the HAC algorithm can meet the requirements. However, considering the time complexity of the HAC algorithm, it is necessary to convert single large data set into multiple small data sets. In the solution of this paper, the data to be clustered has been converted from single big data set to multiple small data sets through the initial allocation, type labeling, and reallocation modules, and the above demand is successfully met.

## 3. Scheduling Problem and Mathematical Model of Garbage Collection and Transportation

In environmental sanitation, the collection and transportation of garbage has its own unique processes. Research on these processes should pay attention to each step and build the model from the business processes, which is also the key to controlling costs.

### 3.1. The Model of Garbage Collection and Transportation

**Figure 2:**
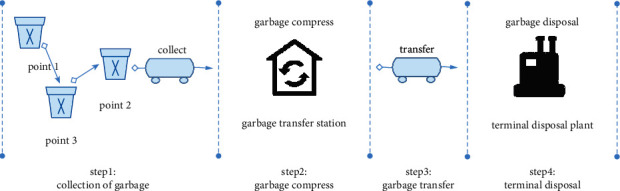
The various processes of garbage collection and transportation.

The various processes of MSW collection and transportation mainly involve three fixed environmental sanitation components, namely, garbage collection point, garbage transfer station, and terminal disposal plant. The processes of garbage collection and transportation are shown in Figure 2, and the main steps are as follows:

- Garbage collection: the collection process is step 1 in Figure 2. The garbage vehicle collects the garbage of each garbage collection point until the amount reaches the vehicle's maximum carrying capacity and then transports the collected garbage to the garbage transfer station. As for the specific garbage transfer station, it depends on manual scheduling in the original operation mode. Each garbage collection point has a garbage production, and this value used in this paper is the average daily garbage production, where the data comes from the production information collected in real-time by the sanitation system.
- Garbage compression: the compression of garbage is step 2 in Figure 2. The garbage vehicles transport the collected garbage to the transfer station for compression.
- Garbage transportation: the transportation process is step 3 in Figure 2. The garbage is transported to the terminal disposal plant for final disposal after being compressed at the garbage transfer station. In the original operation model, the choice of which specific terminal treatment plant to treat waste is related to the operating region, and only the terminal treatment plant corresponding to that area can be selected.
- Terminal disposal: the disposal process is step 4 in Figure 2, mainly for the final treatment of incoming garbage. Each terminal disposal plant limits the amount of garbage that can process per day. Since each region restricts the final disposal plant of garbage, the amount of garbage in the terminal disposal plant is often excessive in the original operation mode. It is necessary to realize the allocation of garbage according to the capacity of the terminal disposal plant.

### 3.2. Mathematical Formulation

Under the divide-regional operation mode, the garbage from each garbage collection point of a street in a certain region can only be transferred to the garbage transfer station of this street and then transported to the terminal disposal plant specified in this region. In contrast, the cross-regional operation scheme does not have the constraint of the region. The garbage from each garbage collection point can be transferred to any transfer stations and terminal disposal plants that meet the disposal capacity. After clustering, the collection and transportation costs of a single garbage collection point cluster in step 1 in Figure 2 are

(1) $$\begin{array}{c} {\text{Cost}}_{i1}=\sum_{j=1}^{k-1}\text{dis}\left(s_{ij},s_{i\left(j+1\right)}\right)+\text{dis}\left(s_{ik},z_{i}\right). \end{array}$$

The transport cost of garbage from the transfer station to the terminal disposal plant is

(2) $$\begin{array}{c} {\text{Cost}}_{i2}=\text{dis}\left(z_{i},c_{i}\right). \end{array}$$

Therefore, the total collection and transportation costs of a single garbage collection point cluster *C*_*i*_ are

(3) $$\begin{array}{c} {\text{Cost}}_{i}=\sum_{j=1}^{k-1}\text{dis}\left(s_{ij},s_{i\left(j+1\right)}\right)+\text{dis}\left(s_{ik},z_{i}\right)+\text{dis}\left(z_{i},c_{i}\right). \end{array}$$

The total collection and transportation costs of all garbage collection point clusters are

(4) $$\begin{array}{c} \text{Cost}=\sum_{i=1}^{K}\left(\sum_{j=1}^{k-1}\text{dis}\left(s_{ij},s_{i\left(j+1\right)}\right)+\text{dis}\left(s_{ik},z_{i}\right)+\text{dis}\left(z_{i},c_{i}\right)\right). \end{array}$$

The total amount of garbage of a single garbage collection point cluster should meet

(5) $$\begin{array}{c} W_{i}=\sum_{j=1}^{k}w_{ij}\leq M. \end{array}$$

The amount of garbage into each terminal disposal plant should meet

(6) $$\begin{array}{c} WP_{h}=\sum_{r=1}^{R_{h}}WC_{r}\leq MP_{h}. \end{array}$$

Therefore, this paper presents the following general mathematical model for dispatching vehicles collecting and transporting garbage. The meaning of each parameter is shown in Table 1.

(7) $$\begin{array}{c} f=\sum_{i=1}^{K}\left(\sum_{j=1}^{k-1}\text{dis}\left(s_{ij},s_{i\left(j+1\right)}\right)+\text{dis}\left(s_{ik},z_{i}\right)+\text{dis}\left(z_{i},c_{i}\right)\right), \end{array}$$

(8) $$\begin{array}{c} \text{s}.\text{t}.\left\{\begin{array}{c} W_{i}=\sum_{j=1}^{k}w_{ij}\leq M,\;\;0\leq i\leq K,\\ WP_{h}=\sum_{r=1}^{R_{h}}WC_{r}\leq MP_{h},\;\;0\leq h\leq H. \end{array}\right. \end{array}$$

Equation (7) defines the objective optimization function of the vehicle driving distance during collection and transportation. Equation (8) ensures that the amount of garbage carried by each garbage vehicle is within its maximum carrying capacity and the amount of garbage entering the terminal disposal plant per day is within its processing capacity.

## 4. Cross-Regional Garbage Collection and Transportation Mode

For reducing the cost of garbage collection and transportation, the following issues should be considered:

- Each garbage collection point should transfer the garbage to the garbage transfer station.
- Each garbage transfer station should transfer the garbage to the terminal disposal plant.
- Determine the optimal driving path of garbage vehicles in each collection point cluster.

The initial allocation, type labeling, and reallocation modules in the cross-regional operation scheme solve the first two problems, while the IHAC and OGCR algorithms solve the last problem.

**Figure 3:**
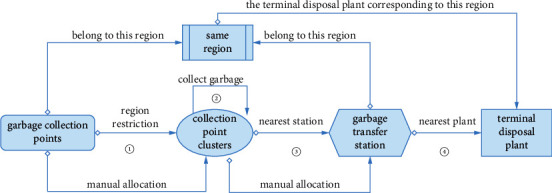
The process of divide-regional operation mode.

In the actual divide-regional operation mode, the processes of garbage collection and transportation are shown in Figure 3, and the detailed steps are as follows:

1. In a certain region, garbage collection points are manually divided into multiple collection point clusters based on geographic location, and this process is restricted by different regions.
2. In a single operation, the garbage vehicle collects garbage from each garbage collection point in a certain garbage collection point cluster, and the collecting route is based on manual scheduling.
3. After the garbage vehicle collects garbage from all garbage collection points in a certain collection point cluster, the garbage vehicle will select the nearby transfer station to compress the garbage according to the manual schedule, usually the nearest transfer station.
4. After the transfer station compresses the garbage, the next step is to transport the garbage to the terminal disposal plant for final disposal. In the divide-regional operation mode, the selection of the disposal plant should be based on the disposal plant list specified in the region, and then the nearest disposal plant that meets the processing capacity will be selected and finally transport the garbage to the designated disposal plant.

There are many constraints in the divide-regional operating mode. Firstly, in the single operation of the garbage vehicle, garbage collection points are manually allocated to form clusters, these collection points may not be clustered geographically, and the collection route of the garbage vehicle is also manually scheduled, which may not be the best collection route, and it will consume many human resources. Secondly, transfer stations for compressed garbage can only be chosen from those in the same region as these collection points, which greatly limits the operating cost. The selection of disposal plants is also limited by the region. The selection of transfer stations and disposal plants is also based on manual work, which consumes many human resources and cannot realize real-time scheduling. This approach also often results in waste not being allocated according to the disposal capacity of the disposal plant, and the amount of waste entering the disposal plant is exceeded.

**Figure 4:**
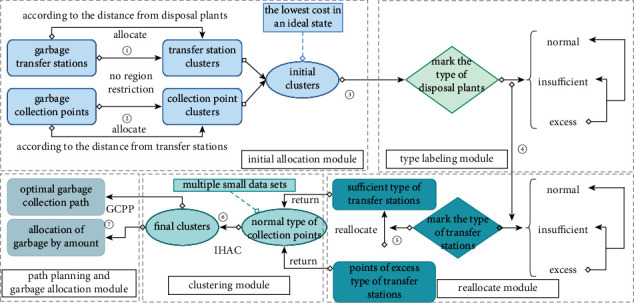
The process of cross-regional operation mode.

For the cross-regional operation scheme, the restrictions of regions are eliminated. From the perspective of a large region, this scheme considers the factors of distance and the amount of garbage, converts single large data set into multiple small data sets through the initial allocation, type labeling, and reallocation modules, and uses the IHAC algorithm and the GCRO algorithm to realize the allocation of garbage according to the amount (allocated according to the disposal capacity of the disposal plant) and the intelligent path planning of garbage vehicles. The process of the cross-regional operation mode is shown in Figure 4, and the detailed steps are as follows:

1. Each transfer station is allocated to the nearest disposal plant based on the distance from each plant, and transfer station clusters are finally formed.
2. Each collection point is allocated to the nearest transfer station based on the distance from each transfer station, and collection point clusters are finally formed.
3. Obtain the initial clusters from steps (1)-(2), calculate the total amount of garbage for each disposal plant under the initial clusters (the amount of garbage is based on the garbage production of all collection points that transport garbage to the corresponding disposal plant), and then mark the type of each disposal plant according to that value and the actual business settings (excess type: exceed the disposal capacity of the disposal plant, insufficient type: less than 19/20 of the disposal capacity of the disposal plant, and normal type: the amount of garbage is between 19/20 of the disposal capacity of the disposal plant and maximum disposal capacity).
4. According to the disposal plant types marked in step (3), further mark the types of transfer stations allocated to the corresponding disposal plant (excess type, insufficient type, and normal type).
5. Obtain collection points corresponding to the excess type of transfer stations, and reallocate these collection points to the insufficient type of transfer stations according to factors such as distance and garbage amount, so that the insufficient and excess type of disposal plants return to the normal type.
6. After the above steps, the IHAC algorithm is used to cluster all collection points corresponding to each transfer station according to the distance and amount of garbage and obtain the final collection point clusters.
7. Use the GCRO algorithm to obtain the optimal collection path of garbage vehicles.

In the cross-regional operation mode, step (1) and step (2) obtain transfer station clusters and collection point clusters according to the distance, respectively, and form the initial clusters. Since the initial clusters only consider the optimal distance, the operating cost under the initial clusters is the lowest. It does not consider the amount of garbage (i.e., the amount of garbage is not allocated according to the disposal capacity of the disposal plant), which will lead to extreme situations, such as some disposal plants dispose of a small amount of garbage, while some disposal plants dispose of a lot. Step (3) and step (4) mark the type of disposal plant and transfer station according to the amount of garbage into each disposal plant under the initial clusters. In the reallocation module of step (5), the two transfer stations (i.e., the insufficient type of transfer station and the excess type of transfer station) corresponding to the reallocation of collection points are first determined based on the following priorities: (1) prioritizing the two closest transfer stations (i.e., the closer the distance, the lower the cost); (2) prioritizing the excess type of transfer station with a small number of collection points, which can minimize the number of collection point clusters finally formed in the clustering module; (3) prioritizing the corresponding collection points with a high total waste production of the excess type of transfer station, which can avoid too many transfer stations participating in the process of reallocation module (i.e., the more transfer stations/collection points participate in the reallocation, the greater the impact on the lowest cost under the initial clusters). Then, the proposed scheme determines which garbage collection points correspond to the excess type of garbage transfer station for reallocation according to the following priorities: (1) prioritizing the garbage collection point closest to the excess type of transfer station; (2) prioritizing the garbage collection point with high garbage production, which can minimize the number of garbage collection points that need to be reallocated and reduce the impact on the lowest cost under the initial clusters. In the reallocation module, the relevant collection points will be continuously reallocated until the amount of waste into disposal plants return to their disposal capacity (i.e., all excess and insufficient types of disposal plants return to the normal type). In step (6), the number of collection points to be clustered has been converted from all collection points in the whole city to multiple small collection point clusters (i.e., the collection points corresponding to each transfer station) through the previous steps, which realizes the conversion from single big data set to multiple small data sets, and provides conditions for the use of hierarchical agglomerative clustering algorithm with time complexity of O(n^3^). The improved hierarchical agglomerative clustering (IHAC) algorithm is formed by improving the HAC algorithm. In the IHAC algorithm, by considering the distance and the maximum carrying capacity of the garbage vehicle, collection points from each collection point cluster are clustered again to obtain the final collection point clusters, where the collection points are relatively clustered geographically and the total amount of garbage is close to the maximum carrying capacity of the garbage vehicle. In step (7), the garbage collecting path planning (GCPP) algorithm is used to plan the garbage collecting path of the garbage vehicle for collecting the garbage from each final collection point cluster, and the optimal collecting path of the garbage vehicle is obtained.

## 5. Algorithms for the Model

### 5.1. The Improved Hierarchical Agglomerative Clustering Algorithm

One of the focuses of this paper is the clustering of collection points, which is based on the distance and the amount of garbage. From the perspective of the amount of garbage, firstly, the total amount of garbage in a single operation (i.e., a collection point cluster) of a garbage vehicle should be controlled within the garbage vehicle's maximum carrying capacity and as close as possible to its maximum carrying capacity, so as to achieve the maximum utilization of the garbage vehicle resources. Secondly, the total amount of garbage transported to the corresponding disposal plant should be within the maximum processing capacity, which can avoid excessive amounts of garbage entering the disposal plant. Based on the above purposes and determining the optimal scheduling scheme of garbage vehicles, this paper improves the HAC algorithm and proposes an improved hierarchical agglomerative clustering (IHAC) algorithm. After the initial allocation, type labeling, and reallocation modules, all the collection points corresponding to each transfer station are clustered again.

The traditional HAC algorithm only takes the distance between the two clusters as the judgment basis for measuring similarity and merges the two nearest clusters. The definition of similarity is relatively simple. The IHAC algorithm proposed in this paper improves the similarity measurement combined with the research scenario: (1) Based on considering the optimal distance, the amount of garbage is considered, and the total amount of garbage of the two merged subclusters cannot exceed the maximum carrying capacity of garbage vehicles. (2) The operating cost of the merged cluster is calculated in advance, which cannot be greater than the sum of the operating costs of the two subclusters, and only the merging that brings cost optimization will be considered. On the termination condition of the iteration, the traditional HAC algorithm needs to set the termination conditions of iteration, which is generally terminated by the percentage of the number of clusters in the original number of clusters. It is a subjective setting and requires certain prior knowledge. Since the IHAC algorithm introduces additional indicators in the measurement of similarity, it does not need to set any iterative termination conditions and only stops the merging operation when no merging pair *P* satisfies any conditions.

In Algorithm 1, the input Ids, locations, and weights correspond to the unique number of the collection points, latitude and longitude, and garbage production, and the output is the final clusters. Firstly, each collection point is initialized as a cluster, clus represents the final clusters, cluWts represents the total amount of garbage in each cluster, and CP*s* represents the center of each cluster (i.e., the average of longitude and latitude of all points in the cluster). Secondly, the distance matrix *C* is calculated. The distance between the two clusters is calculated based on the center of the cluster CP*s*. Using the above method as the criterion for measuring distance can avoid the error caused by singular points. Row 6 in Algorithm 1 means calculating the distance between any two clusters. Rows 7 to 18 obtain the two subclusters that can be merged. According to the distance matrix, row 8 obtains the two nearest subclusters *X* and *Y*. Row 12 represents calculating the operating cost of the cluster *Z* formed by the merging of *X* and *Y* in advance (the operating cost includes the whole process of garbage collection and transportation, i.e., equation (3)). Rows 14 to 15 represent calculating the operating cost of the two subclusters, respectively. Row 16 indicates the merging conditions: (1) The operating cost of the merged cluster is less than the sum of the operating costs of the two subclusters. (2) The total amount of garbage in the two subclusters cannot be greater than the maximum carrying capacity of the garbage vehicle. The two subclusters that meet these merging conditions become the merging pair *P*. Otherwise, continue to judge the next subcluster pair according to the distance matrix *C* from near to far. Rows 19 to 30 represent the process of iterative merging subclusters and updating relevant variables. When a cluster pair P satisfies the merging condition, row 22 represents the merged cluster *Z* that replaces the original subcluster *X*, and the subcluster *Y* is removed. As shown in row 24, since the members of the cluster *X* have changed, it is necessary to recalculate the center point of cluster *X* and remove the center point information CP*s* [*Y*] of the subcluster *Y*. As shown in row 26, since the subcluster *Y* is merged into the subcluster *X*, the total garbage production of the cluster *Y* should be added to the total garbage production of the cluster *X*, and the garbage production information of the cluster *Y* should be removed. Finally, the distance matrix *C* is updated according to the new center point information CP*s*. The merging operation is repeated until the *P* is empty and the final collection point clusters are returned.

### 5.2. The Garbage Collecting Route Optimization Algorithm

For each garbage collection point cluster finally obtained from the IHAC algorithm, the key to controlling the operating cost is to plan the collecting path of garbage vehicles. Since the total amount of garbage in each garbage collection point cluster is controlled within the maximum carrying capacity of the vehicle after the IHAC algorithm, the number of collection points in each cluster is in a small order of magnitude, and the greedy search idea can be used to search the optimal collecting route of the garbage vehicle. Searching the optimal collecting path (i.e., the driving path of garbage vehicles collecting garbage) can be regarded as a problem with multiple starting points and a single ending point. The starting point can be any collection point in the cluster, and the ending point can only be the transfer station corresponding to the cluster.

In Algorithm 2, the operating cost of the garbage vehicle in a single operation (i.e., a single collection point cluster) includes the driving distance between each collection point and the driving distance between the last collection point and the transfer station. As shown in row 3, Algorithm 2 first uses the idea of greedy search to obtain all possible collecting paths for garbage vehicles. Rows 4 to 11 calculate the operating cost of all paths. Row 7 calculates the driving cost between collection points, and row 9 calculates the driving cost from the last collection point to the transfer station. Row 13 obtains the path with the lowest operating cost path according to the respective costs of all possible paths.

In Algorithm 2, GCPList represents the garbage collection point list, *GTSLoc* represents the longitude and latitude of the transfer station, and nums represents the number of collection points. The output is the optimal collecting path of these collection points. The total collection and transportation cost of each collection point cluster includes the collection and transportation cost between collection points and the collection and transportation cost from the last collection point of the cluster to the transfer station corresponding to the cluster. Algorithm 2 lists all possible collection routes by the permutation module (since the IHAC algorithm controls the number of collection points in each cluster, this search for the optimal collecting route is feasible) and calculates the distance of these routes (the distance between the collection points and the distance between the collection point and the transfer station). Algorithm 2 will select the route with the lowest distance to obtain the optimal vehicle collection route. The pseudocode of the GCPP algorithm is as follows.

**Figure 5:**
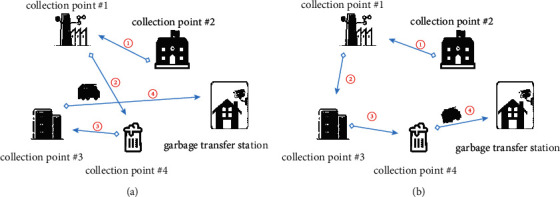
Collection route of garbage vehicle before and after optimization.

The planning effect of Algorithm 2 on the optimal collecting path of garbage vehicles is shown in Figure 5.  Figure 5(a) shows the collecting path before the planning, and Figure 5(b) shows the optimal collecting path after the planning.

## 6. Simulation Experiment and Discussion

### 6.1. Experiment Data and Evaluation Criteria

**Figure 6:**
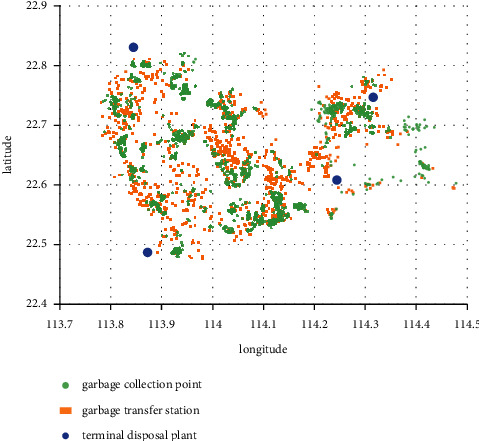
Visualization of data.

The data of the experiment comes from the actual environmental sanitation data of X city in 2021, including 2182 garbage collection points, 940 garbage transfer stations, and 4 terminal disposal plants. The relevant information of garbage collection point includes latitude and longitude, daily garbage production, region, and street. The relevant information of the garbage transfer station includes latitude, longitude, region, and street. The relevant information of the terminal disposal plant includes latitude and longitude, daily maximum disposal amount, and accepted regions. Detailed data information is shown in Table 2. The geographic location of each sanitation component is visualized, as shown in Figure 6.

In the collection and transportation of garbage, some cost evaluation criteria in this field include the driving distance of garbage vehicles, the number of garbage vehicles put into use, and the consumption of human resources. The driving distance of garbage vehicles usually embodies working time and fuel consumption. In the development of green cities currently, it is particularly important to reduce fuel consumption in carbon neutralization. Due to the lack of relevant road network data, the experiment in this paper uses the linear distance between the latitude and longitude of the garbage collection points to measure the driving distance. After many simulation experiments, we find that this linear distance can accurately evaluate cost optimization in the case of large-scale data. The number of garbage vehicles put into use is related to whether the garbage vehicle resources put into use are fully utilized. The standard of human resource consumption is used to evaluate whether the scheme is automated rather than based on huge human consumption. As for whether the allocation of waste is scientific and reasonable, the main evaluation criterion in this field is to determine whether the amount of waste disposed of by each facility conforms to the design of its disposal capacity. For example, the amount of waste entering the terminal disposal plant should not exceed its maximum disposal capacity. Therefore, the design of the experiment is mainly carried out from the above evaluation criteria.

### 6.2. Comparative Experiment

Based on the above evaluation criteria, this paper sets up multiple comparative experiments to compare and analyze the new method proposed in this paper with other similar advanced methods:

- The divide-regional operation scheme (i.e., the scheme adopted by the city X currently, denoted as method 1). In method 1, the various processes of garbage collection and transportation are limited by the region, and the garbage collecting paths of garbage vehicles are based on the manual arrangement. Since each operational process has been immobilized in method 1, no parameters need to be set. For the fairness of the experiment, this work uses the same evaluation method in this paper to evaluate the performance indicators of method 1.
- A simulated annealing (SA) algorithm for planning the collecting path of garbage vehicles in the context of MSW (denoted as method 2) proposed by M. Fermani et al. [35]. A simulated annealing algorithm is a classical stochastic optimization algorithm for solving the CVRP, and it is representative. Method 2 applies it to the field of garbage collection and makes optimization, so this work takes method 2 as one of our comparative experiments. On the parameter setting, the function of the cooling schedule is T(*t*)=*αT*(*t* − 1), the cooling factor *α*=0.9, the initial temperature *T*_0_=100, the initial number of garbage vehicles *v*_*n*_=*W*/*M*, where *W* is the total garbage production and *M* is the maximum carrying capacity of the garbage vehicle, the number of iterations *N*_*t*_ = 200 performed by the algorithm for a certain temperature value *T* in iteration *t*, the maximum allowable number of iterations without improvement *N*_stop_=2000, and the probability of mutation *p*_*m*_=0.25. Since the simulated annealing algorithm is a random algorithm, the results are not unique; this work conducted 50 tests and took the optimal value in the results.
- A method KMeans-Dijkstra (denoted as method 3) based on recursive k-means clustering and the Dijkstra algorithm proposed by Moussa [36]. The idea of method 3 is similar to that of the method in this paper. It uses the recursive k-means clustering to form clusters that meet the capacity of vehicles, and then it uses the Dijkstra algorithm to find the optimal access path of the points in each cluster. It is a novel method in this field and similar to the method in this paper, so this work takes it as a comparative method. In method 3, it introduces the occupancy rate of the cluster (i.e., the percentage of the total amount of garbage in the cluster to the maximum carrying capacity of the vehicle in the scenario of this paper). A cluster belongs to a low occupancy cluster when its occupancy rate is below a threshold. The points in the low occupancy cluster need to be assigned to the cluster corresponding to the nearest centroid under the constraint. Due to the constraint of k-means clustering, the number of clusters *k* needs to be set in advance, but we do not have a priori knowledge of this value. Therefore, when conducting experiments of this method, this work initializes the value of *k* to the optimal value and iterates by increasing this value. On the parameter setting, the number of initial clusters *k*=*W*/*M*, and the threshold of low occupancy rate *q*  *=* 0.2. Since the k-means algorithm in the initial centroid selection is random and the results are unstable, this work conducted 50 tests and took the optimal results.
- A method PVNS-ASM (denoted as method 4) proposed by Faiz et al. [37]. Method 4 is an enhanced perturbation-based variable neighborhood search with an adaptive selection mechanism. On the parameter setting, this work adopts the optimal configuration of method 3 in [37], the best configuration of the shaking step: N1: exchange (m, n), N2: cross, and N3: shift (m, 0) and the best configuration of the local search step: N1: insertion, N2: exchange, N3: 3-opt, N4: 2-opt, N5: replace, N6: shift, and N7: cross; that is, *k*_max_ is set to 3 and *m*_max_ is 7, the perturbation counter *p*_max_ is 50, the VNS termination counter *s*_max_ is 100, and the parameter of scoring system *θ*_1_ is set to 5 and *θ* is 10. This work conducted 50 tests of this method and finally took the optimal value of the results.

In methods 2 to 4, for experimental comparability, the initial data (i.e., each garbage transport station and all garbage collection points corresponding to it) comes from the garbage collection point clusters formed after the initial allocation, type labeling, and reallocation modules. The research scenario of methods 2 to 4 is slightly different from this paper's research scenario. For the fairness of the experiment, some modifications are made: the ending point can only be the garbage transfer station, and the starting point is changed from the fixed point to any garbage collection points (i.e., the starting point can be any garbage collection point corresponding to garbage transfer station). The comparison between method 1 and our method can illustrate the advantages of the cross-regional operation model. The comparisons of methods 2–4 with our method can illustrate the advantages of the IHAC algorithm and the GCPP algorithm.

### 6.3. Analysis of Experimental Results

The experimental results are shown in Table 3. The average weight refers to the average weight of garbage carried by each garbage vehicle in its single collecting work. The utilization of vehicle capacity refers to the average rate of garbage carried by vehicles to their capacity in a single operation. The runtime of an algorithm is the average runtime of that algorithm. The total driving distance of all garbage vehicles is calculated by latitude and longitude between geographical locations. The rows 1–5 of Table 3 correspond to methods 1 to 4 and the methods in this paper, respectively.

The results show that the proposed cross-regional operation scheme is superior to other comparison methods in terms of the driving distance of vehicles, the number of garbage vehicles, the utilization rate of garbage vehicle resources, and the algorithm running time. Method 1 does not involve the running time of the algorithm because it does not involve any algorithm in method 1 and only calculates other evaluation criteria according to the work schedule in the actual operation. From the comparison of method 1 with other methods, the results show that the great advantages brought by the cross-regional operation model proposed in this paper have greatly improved the performance of each key performance indicator.

**Figure 7:**
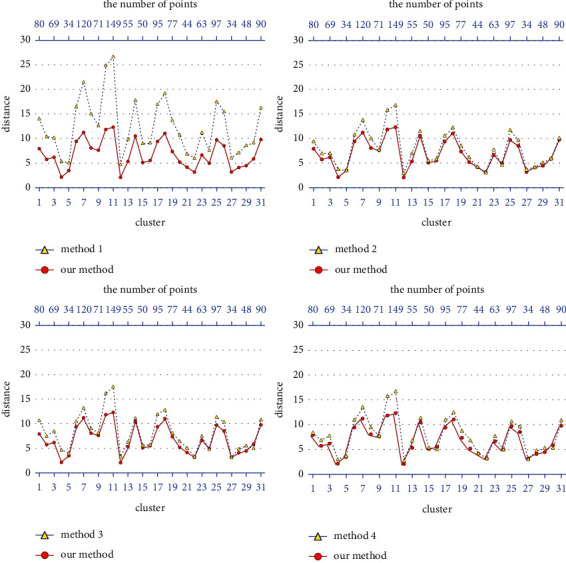
The driving distance performance of various methods on different clusters.

The performance of driving distances of different methods on different clusters is shown in Figure 7. The *x*-axis below in Figure 7 represents different clusters. After the initial allocation, type labeling, and reallocation modules, there are 31 garbage collection points clusters. The *x*-axis above in Figure 7 represents the number of points in each cluster. The *y*-axis represents the total driving distance of garbage vehicles in each cluster. The sum of garbage vehicles' driving distances in each cluster is the total driving distance of garbage vehicles in the corresponding method in Table 3. According to the comparison results between various methods and our method in Figure 7, our method IHAC-GCPP is superior to other methods in terms of the driving distance of garbage vehicles. When the number of points in each cluster is greater, the optimization of the method IHAC-GCPP is larger. In method 1, the various processes of garbage collection and transportation are inflexible due to the constraint of the region, and garbage vehicles can only travel some additional distances to complete the operation according to the provisions of the region, so the performance of garbage vehicles in the driving distance is relatively poor. The respective total driving distance of garbage vehicles in the SA and the PVNS-ASM is slightly longer than that of the IHAC-GCPP. In the method SA, the setting of the cooling value *α* affects the final results of the simulated annealing algorithm. If the cooling speed is too fast, searching for a better solution may be faster, but it may also skip the optimal solution directly. If the cooling speed is too slow and the search time is too long, a better solution can be obtained, but it will take much time. The setting of iterations in the simulated annealing algorithm is similar to this situation. The method PVNS-ASM combines PVNS and ASM and uses a simple perturbation strategy to expand the search horizon for producing a more robust solution. However, sometimes due to the premature convergence of the search process, the algorithm cannot continue the search process to another promising region. In the method KMeans-Dijkstra, since the garbage collection points of the low occupancy cluster will be allocated to the other appropriate clusters, an additional driving distance will be brought. Therefore, the total driving distance of garbage vehicles in the KMeans-Dijkstra is longer than that in the SA and the PVNS-ASM. The results show that, compared with the current application method (i.e., the divide-regional operating method) in city X, the total driving distance of all garbage vehicles is reduced by 44.387% in the IHAC-GCPP; that is, the total operating time/fuel consumption of garbage collection and transportation is reduced by 44.387%, 12.099%, 14.508%, and 11.230%, respectively, compared with methods 2, 3, and 4.

**Figure 8:**
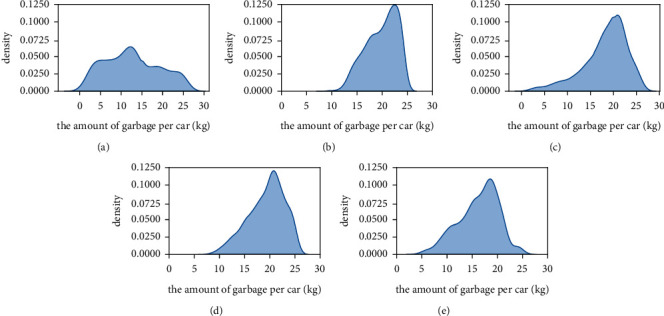
The distribution of garbage carried by garbage vehicles in each method. (a) Method 1. (b) Our method. (c) Method 2. (d) Method 3. (e) Method 4.

**Figure 9:**
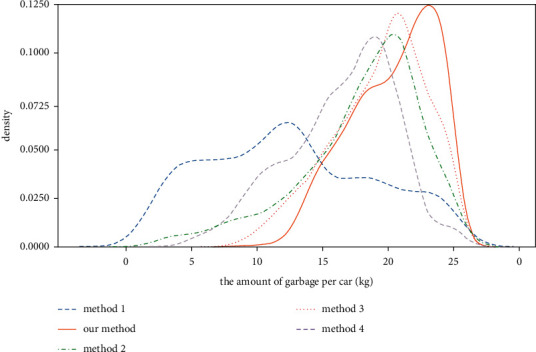
Comparison of the distribution of garbage carried by garbage vehicles in each method.

The distribution of garbage carried by garbage vehicles in each method is shown in Figure 8, and the comparison between them is shown in Figure 9. The garbage vehicles used in method 1 cannot fully use their carrying capacity, resulting in this result related to the unreasonable manual scheduling of garbage vehicles and the constraint of operating regions. In the KMeans-Dijsterla, due to the introduction of the occupancy rate *q*, the garbage collection points of the low occupancy cluster will be allocated to the other appropriate clusters. It can be seen that there is no case of low garbage amount carried by garbage vehicles in the KMeans-Dijkstra from Figure 8(d), which is only slightly lower than the IHAC-GCPP in this paper in the utilization rate of vehicle resources. However, it has an additional impact on the vehicle's driving distance. It can be seen from the results that the performance is poor in terms of the driving distance of the vehicle. In the IHAC-GCPP, the IHAC algorithm will consider the distance and the amount of garbage in the clustering process and cluster according to a certain priority. Therefore, it will not increase the driving distance of the vehicle while minimizing the number of vehicles. At the same time, the GCPP algorithm uses a greedy strategy to search for the optimal driving path of the vehicle. Due to the small number of samples of each cluster after clustering, the GCPP algorithm shows high efficiency. The results show that, compared with the divide-regional operating method, the number of garbage vehicles put into use decreased by 36.107% in the IHAC-GCPP, and the utilization rate of vehicle carrying capacity is increased by 64.614%. In the original garbage collection and transportation operation, the operating paths of garbage vehicles need many human resources to plan. The method proposed in this paper realizes the automatic scheme of allocating garbage according to the amount of garbage and the optimal operating path of garbage vehicles and realizes zero human consumption.

**Figure 10:**
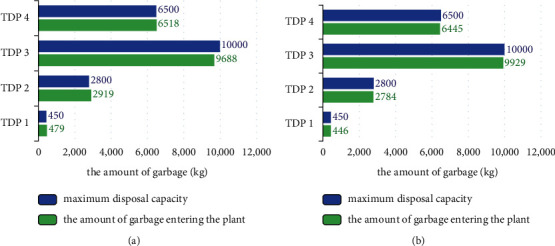
Comparison of garbage amount entering the plant before and after optimization.

One of the problems to be solved in this paper is the excess amount of garbage entering the disposal plant. In the divide-regional operating method, the events that the amount of garbage entering the terminal disposal plant exceeds its maximum disposal amount often occur due to each region stipulating which plants the waste is transported to for disposal. The cross-regional operation scheme can allocate garbage flexibly according to the disposal capacity of the terminal disposal plant and ensure that the amount of garbage entering each plant is within its disposal capacity.  Figure 10 takes such an above event in city X as an example to compare the amount of garbage entering each plant in the divide-regional operating method and cross-regional operating method.  Figure 10(a) represents the situation of a certain garbage excess event in the divide-regional operation model, and it can be found that the garbage in some disposal plants exceeds its maximum disposal capacity, which is mainly caused by the constraint of the region. Since each region stipulates that the garbage should be transported to which disposal plants for processing, it is not suitable for the amount of garbage that is dynamically changing every day.  Figure 10(b) shows how the above garbage excess event is solved in the cross-regional operation model. Since the cross-regional operation scheme eliminates the constraint of the region, it can be seen that this problem has been well solved under this scheme. For the garbage amount that is dynamically changing every day, the scheme will allocate the garbage according to the garbage amount that is dynamically changing and the disposal capacity of the terminal disposal plant.

According to the experimental results and comparative analysis, the method IHAC-GCPP is superior to other methods in the key performance indicators of garbage collection and transportation. For one thing, the operating distance of garbage vehicles and the number of garbage vehicles put into use are greatly reduced, which is lower than other methods. The utilization rate of vehicle carrying capacity is also the best, which greatly reduces the cost of garbage collection and transportation, and saves many material resources to be invested. At the same time, the IHAC-GCPP realizes the intelligent scheduling of vehicle operating routes and forms an automatic scheduling scheme. It can liberate vehicle dispatchers from daily tedious scheduling work, realize zero consumption of human resources in vehicle scheduling, and save many human resources to be invested. For another, in the allocation of garbage, the cross-regional operation scheme comprehensively considers the distance and the maximum disposal capacity of the terminal disposal plant to flexibly allocate garbage so as to ensure that the amount of garbage entering each disposal plant does not exceed its maximum disposal capacity. The experimental results show no longer an excess event of garbage amount in the cross-regional operation scheme, which realizes the allocation of garbage according to disposal capacity.

## 7. Conclusions

In this paper, the path planning of garbage collection and transportation and the optimization of garbage allocation in the environmental sanitation work are studied. This work analyzes the constraints of current garbage collection and transportation schemes in practical application, establishes a mathematical model considering the driving distance and garbage amount constraints, and proposes a new cross-regional operation scheme. In the experiment, in order to evaluate the effectiveness of the proposed scheme, the evaluation index system of garbage collection and transportation in sanitation operations is established, and comparative experiments are carried out to compare and analyze the method in this paper and other advanced methods.

The real operation data of city X in sanitation work is used as the data set to conduct the comparative experiment. The results show that the proposed scheme greatly reduces the driving distance of vehicles in garbage collection and transportation and the number of garbage vehicles put into use and greatly improves the utilization rate of the vehicle carrying capacity. The IHAC algorithm and GCPP algorithm can obtain better solutions and convergence speed. The method IHAC-GCPP enables the intelligent route planning of vehicles in garbage collection and transportation and the allocation of garbage according to the disposal capacity, and garbage excess events no longer occur. It can be seen from the comparison of the results that, in the established evaluation index system, our method IHAC-GCPP is superior to other methods in the performance of each evaluation index.

This work is helpful to promote innovation in the field of garbage collection and transportation. It can guide urban sanitation departments and provide decision support for scientific and intelligent collection and transportation of garbage.

This paper took the related factors such as distance and garbage amount constraints into account in the garbage collection and transportation planning model. However, due to the lack of road network data and road feature data, the actual road distance is not applied when considering the driving distance, some of which have not been considered further, such as road traffic state. In future work, these factors can be considered in the model to improve the robustness of the model further.

## Figures and Tables

**Algorithm 1:**
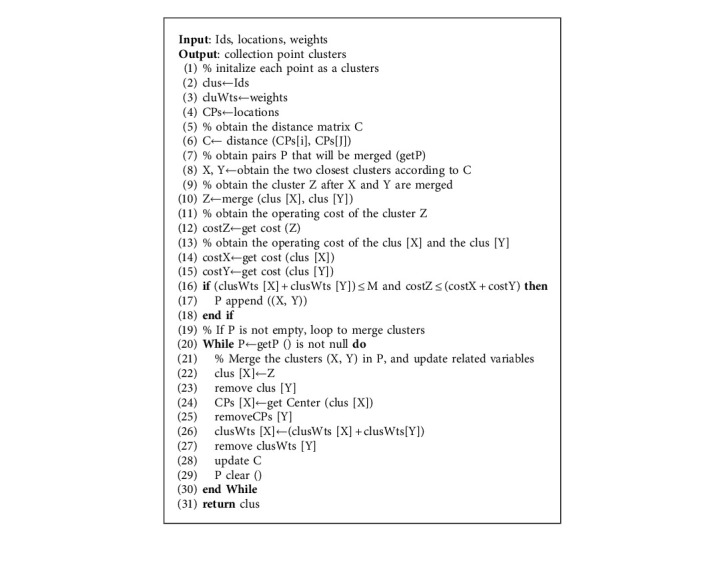
Improved hierarchical agglomerative clustering (IHAC) algorithm.

**Algorithm 2:**
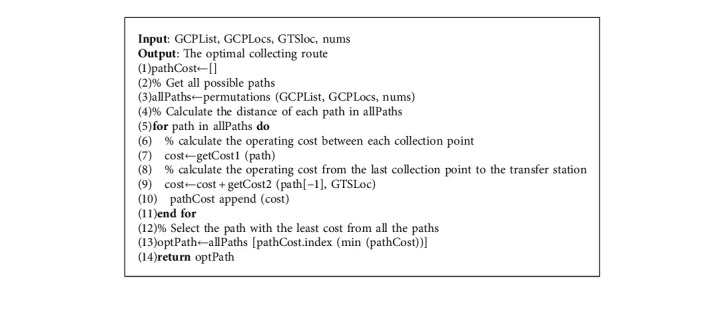
Garbage collecting path planning (GCPP) algorithm.

**Table 1:** The meaning of each symbol.

Parameter	Meaning
*k*	Number of collection points in the *i*-th garbage collection point cluster
*K*	Number of garbage collection point clusters
*H*	Number of terminal disposal plants
*C*_*i*_	The *i*-th collection point cluster
*z*_*i*_	The garbage transfer station corresponding to *C*_*i*_
*s*_*ij*_	The *j*-th garbage transfer station in *C*_*i*_
*c*_*i*_	The terminal disposal plant corresponding to *C*_*i*_
*w*_*ij*_	The garbage amount of the *j*-th collection point in *C*_*i*_
*M*	The maximum nuclear load of the garbage vehicle
*WP*_*h*_	The amount of garbage into the *h*-th disposal plant
*R*_*h*_	The number of collection points corresponding to the *h*-th disposal plant
*WC*_*r*_	The total amount of garbage of *C*_*r*_
*MP*_*h*_	The upper limit of the *h*-th disposal plant

**Table 2:** The format of the data set.

Facility	Number	Data format
Garbage collection point	2182	{Latitude, longitude, region, street, daily garbage production}
Garbage transfer station	940	{Latitude, longitude, region, street}
Terminal disposal plant	4	{Latitude, longitude, maximum garbage amount, accepted regions}

**Table 3:** Experimental results of various methods.

Method	Total driving distance	Number of vehicles	Average weight (kg)	Utilization of vehicle capacity	Algorithm runtime (s)
Method 1	389.281	1526	12.943	0.51772	—
SA [35]	246.288	995	19.852	0.79408	235.25
KMeans-Dijkstra [36]	253.229	981	20.387	0.81548	412.317
PVNS-ASM [37]	243.877	989	19.096	0.76384	341.05
IHAC-GCPP	216.490	975	21.306	0.85224	56.985

## Data Availability

Data will be made available upon request to the corresponding author.
